# 4220 Cognitive Function and Relationships with Intervention Dropout, Adherence and Weight Loss

**DOI:** 10.1017/cts.2020.115

**Published:** 2020-07-29

**Authors:** Amanda Nicole Szabo-Reed, Richard Washburn, Laura Martin, Cary Savage, Joseph Donnelly

**Affiliations:** 1 University of Kansas Frontiers; 2 University of Kansas Medical Center; 3 University of Nebraska-Lincoln

## Abstract

OBJECTIVES/GOALS: Greater cognitive function (CF) is associated with adherence to prescription medications, better program adherence and weight loss (WL) following bariatric surgery. The purpose of this study was to evaluate the association between baseline CF, intervention dropout, adherence and 3-month WL. METHODS/STUDY POPULATION: 107 (*Mage* = 40.9 yrs.), overweight/obese (*BMI* = 35.6 kg/m^2^) men (N = 17) and women (N = 90) completed a 3-mo. WL intervention. Participants were asked to attend weekly behavioral sessions, comply with a reduced calorie diet and complete 100 min of moderate intensity physical activity (PA)/wk. CF tasks including Flanker (attention), Stroop (Executive control) and working memory, body weight and cardiovascular fitness (covariate) were assessed at baseline and 3-mos. Session attendance, adherence to PA and diet prescriptions and number of off-diet episodes were recorded weekly. RESULTS/ANTICIPATED RESULTS: Results indicated that attention was positively correlated with session attendance (*p* = 0.016), adherence to the diet (*p* < 0.01) and PA (*p* = 0.023). Executive control was positively correlated with WL (*p* = 0.042). Working memory (two tasks) was also positively correlated with WL (*p* = .017 and *p* = .025). Analysis of variance (ANOVA) indicated that baseline attention (*p* = .012) was positively related to WL and negatively associated with drop out (*p* < .05). Hierarchical linear regression showed executive control (*p* = .036, R^2^ = .054) and working memory (*p* = .013, R^2^ = .073 and *p* = .017, R^2^ = .068) were associated with WL when controlling for fitness. DISCUSSION/SIGNIFICANCE OF IMPACT: These results suggest that stronger baseline attention is associated with completion of a 3-mo. WL intervention. Executive control and working memory are associated with amount of WL achieved. Additional, larger and longer trials to assess the role of baseline CF on WL and evaluating the impact of interventions designed to improve CF on WL are indicated.

